# Etiology and Outcomes of ARDS in a Rural-Urban Fringe Hospital of South India

**DOI:** 10.1155/2014/181593

**Published:** 2014-02-10

**Authors:** Tarun George, Stalin Viswanathan, Ali Hasan Faiz Karnam, Georgi Abraham

**Affiliations:** ^1^Department of Internal Medicine, Pondicherry Institute of Medical Sciences, Kalapet, Pondicherry 6050014, India; ^2^Department of General Medicine, Indira Gandhi Medical College & RI, Kadirkamam, Pondicherry 605009, India

## Abstract

*Objectives.* Etiology and outcomes of acute lung injury in tropical countries may be different from those of western nations. We describe the etiology and outcomes of illnesses causing acute lung injury in a rural populace. *Study Design.* A prospective observational study. *Setting.* Medical ICU of a teaching hospital in a rural-urban fringe location. *Patients.* Patients ≥13 years, admitted between December 2011 and May 2013, satisfying AECC criteria for ALI/ARDS. *Results.* Study had 61 patients; 46 had acute lung injury at admission. Scrub typhus was the commonest cause (7/61) and tropical infections contributed to 26% of total cases. Increasing ARDS severity was associated with older age, higher FiO_2_ and APACHE/SOFA scores, and longer duration of ventilation. Nonsurvivors were generally older, had shorter duration of illness, a nontropical infection, and higher total WBC counts, required longer duration of ventilation, and had other organ dysfunction and higher mean APACHE scores. The mortality rate of ARDS was 36.6% (22/61) in our study. *Conclusion.* Tropical infections form a major etiological component of acute lung injury in a developing country like India. Etiology and outcomes of ARDS may vary depending upon the geographic location and seasonal illnesses.

## 1. Introduction

Acute lung injury/acute respiratory distress syndrome (ALI/ARDS) is a spectrum of inflammatory lung injury often seen in critically ill patients causing hypoxemic respiratory failure. Ashbaugh and colleagues first identified ARDS in 1967 when they described 12 patients with acute respiratory failure refractory to oxygen therapy, who had bilateral chest infiltrates and reduced lung compliance [1]. The etiology of ARDS can be divided into pulmonary (direct) and extra-pulmonary (indirect) causes. Infective and aspiration pneumonia are the commonest pulmonary causes of ARDS, whereas systemic sepsis is the predominant extrapulmonary cause of acute lung injury [2]. Both medical and surgical problems contribute towards ARDS. Medically, a significant proportion of cases in tropical climes are likely to be the result of problems common in the rural tropics: infections such as malaria, scrub typhus, enteric fever, and leptospirosis or injuries gained by poisoning, near hanging, and near-drowning. Inequalities among various ethnic groups are known with respect to mortality rates in ARDS [3]. Mortality rates in the North Indian and Western Indian population were 47.8% [2] and 57% [4], respectively. This study aimed to study the medical causes, outcome, and prognostic factors in patients admitted with acute lung injury in an intensive care unit (ICU) of a rural-urban fringe of South India.

## 2. Materials and Methods

This was a cross-sectional prospective observational study conducted in the medical ICU of the Pondicherry Institute of Medical Sciences between December 2011 and May 2013. This teaching hospital is located on a rural landscape 20 kilometers from the nearest town, Pondicherry, South India. Patients with ALI/ARDS were identified through a prospective daily ICU surveillance, based on the American-European Consensus Conference criteria (AECC). Consecutive patients above 13 years of age with a medical diagnosis leading to ALI/ARDs were studied. Patients with burns, trauma, and postoperative status were excluded. Patients with ALI/ARDS were identified based on history, physical examination, chest radiography, and arterial blood gas analysis. All patients had central venous pressure monitoring at admission and echocardiography was performed in all patients during their ICU stay to rule out cardiogenic causes of respiratory distress. Patients were assessed twice during their hospital stay—first, at time of admission and again at the time of discharge from ICU or at the time of death. Baseline characteristics, including comorbidities, history, biochemical and microbiological investigations, initial SOFA scores, and acute physiology and chronic health evaluation (APACHE II) scores, were documented in an Excel sheet. Also, duration of hospital stay, duration of mechanical ventilation, inotrope use, and ultimate hospital outcome were documented at the time of discharge or death. The Ethics Committee of the institute approved the study.

### 2.1. Statistical Analysis

Statistical analyses were performed using IBM SPSS Statistics 20 for Windows. Independent-samples *t*-test was performed for continuous variables and was expressed using the mean ± SD and the median (range and interquartile range (IQR)). Chi square test (or Fischer's exact test) was performed for categorical data. Statistical significance was defined as *P* values <0.05. Univariate analysis was done to determine relative risk of mortality. Those with a significant *P* value were used in a step-wise multivariate logistic regression analysis to obtain predictors of mortality. Using Kaplan-Meier analysis, survival curves were plotted for patients with infective and noninfective etiologies with respect to hospital stay. The difference between the curves was analyzed using the log-rank test.

## 3. Results

During the study period, 61 patients were admitted into the medical ICU with a diagnosis of either ALI or ARDS, directly from the emergency department, or had been transferred from the wards when they required respiratory support. Our subject cohort was relatively young, with a mean age of 41.90 ± 15.35 years and women on an average (36.93) were 10 years younger than men (46.41). Forty-one cases had direct lung injury, with pneumonia due to infections and aspiration contributing to 70% (*n* = 29) of them. Tropical infections including dengue, malaria, leptospirosis and scrub typhus contributed to 30.43% (*n* = 14/46) of infective causes (Table 1). In the infectious group, neutrophilic leukocytosis, and elevated creatinine were more commonly observed in leptospirosis and bacterial infections, whereas significant thrombocytopenia was seen in leptospirosis and malaria. ESR elevation was highest in tuberculosis and increased liver transaminases were consistently seen in *Rickettsial*, *Leptospiral*, and malarial infections. Hypoalbuminemia (<30 g/L) was observed only in *Rickettsial* diseases.

**Figure 1:**
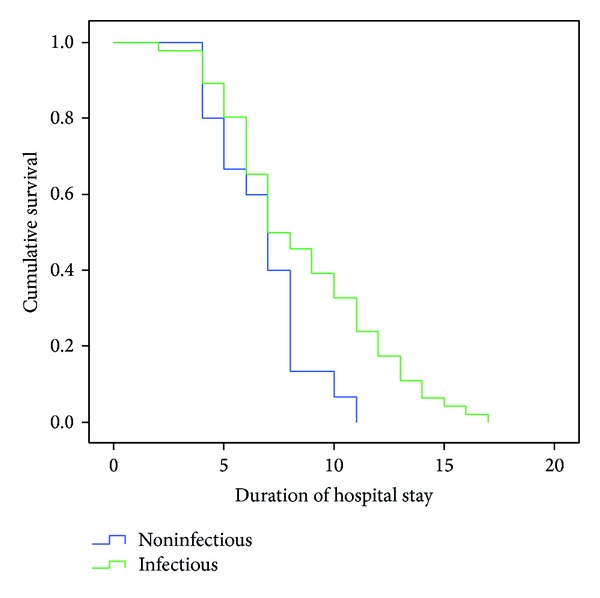
Timing of hospital stay in patients with infectious and noninfectious causes (Kaplan-Meier analysis).

When our study subjects were categorized according to the newer Berlin classification, only four patients had severe ARDS. Increasing severity of ARDS was associated with increases in age, heart rate, FiO_2_, ESR, total leukocyte counts, urea/creatinine, APACHE/SOFA scores, and duration of mechanical ventilation (Table 2). Only 46 subjects (75%) had ARDS at presentation. A final diagnosis of ARDS was made in 38 patients, with 20 succumbing to their illness. The mortality rate in our study was 36% (22/61). Among tropical infections, one case each of scrub typhus and leptospirosis did not survive (2/12), compared to 15 deaths among nontropical infections (15/32). Older age, smoking and alcohol in combination, comorbid conditions like diabetes, and hypertension were significantly related with mortality. Altered sensorium, inotrope use, CPR, and APACHE II score >20 had the highest relative risk of mortality. Multiple logistic regression analysis was performed using significant variables obtained during univariate analysis (Table 3). Only altered sensorium (*P* = 0.011) and inotrope use (*P* = 0.027) were significantly associated with mortality. Initial SOFA scores were significantly (*P* < 0.001) higher in nonsurvivors compared to survivors. The median hospital stay in patients with an infective etiology was 7.5 days (IQR 5 days; range 2 to 17 days) versus 7 days in patients with a noninfective etiology (IQR 3 days; range 4 to 11 days). The difference between the survival curves was analyzed using the log-rank test and was found to be statistically significant (*P* = 0.034) (Figure 1).

## 4. Discussion

Our study had focused on the etiology of ALI/ARDS and prognostic factors in such patients. As this study began before the Berlin Definition came into use, the AECC criteria had been used to enroll and classify our patients [5]. A male predominance in our study was similar to both national and international studies described previously [2, 6–8]. Mean ages of our study subjects were 46.41 ± 13.44 and 36.93 ± 16 years in men and women, respectively. This was closer to that reported from studies in North India and Western India [2, 4] and contrasted the studies by Estenssoro et al., Zilberberg et al., and the Irish Critical Care Trials group where the means were 55 ± 19, 51 ± 2, and 58 ± 17, respectively [7–9]. This could be partially explained by the variation in the age demographics among ICU admissions in our setup. Bhadade et al. described that young men involved in work where environmental exposure to vectors of tropical illnesses is high contributed to the male predominance and younger age of patients in their study of ARDS [4]. Younger patients with noninfectious etiologies like poisoning, pancreatitis, near hanging and near-drowning, and infections such as dengue, scrub typhus, and H1N1 influenza probably contributed to the lower mean age in our study. We did not record occupational history of our patients.

The commonest cause of direct lung injury in our study was infective and aspiration pneumonia, while systemic sepsis and acute pancreatitis were the main contributors of indirect lung injury, a finding shown in many studies [2]. Infections in our study were thrice as common as noninfectious causes of ARDS. A higher proportion of patients (78.%) with moderate ARDS had an infectious etiology. As a group, bacteria, rickettsiae, and viruses were the commonest infections causing ARDS with 11, 7, and 7 cases each, respectively. Pneumonia is the commonest underlying condition of ARDS [10]. *Streptococci pneumonia*, *Staphylococcus aureus*, *Mycoplasma*, *Coxiella*, and Gram-negative bacilli are the common etiological agents of pulmonary infections that cause ARDS and the spectrum of isolated pathogens are similar in America and Europe [10, 11]. Among six patients with community acquired pneumonia (CAP), only one case of *S. pneumonia* could be identified in our study. The etiology of CAP may vary depending on the geographical area and that the microbiology of one-third of cases may remain unidentified [11]. *Pseudomonas aeruginosa* and *Acinetobacter baumannii* were the commonest causes of hospital acquired pneumonia in our study. Since ventilator associated pneumonia (VAP) may complicate the course of ARDS that required mechanical ventilation [10], some primary causes of ARDS may have been unidentified in the cases of VAP seen in our study.

Acute respiratory failure and ARDS are one of the commonest complications of H1N1 infection requiring ICU care. Incidence of ARDS among patients infected with H1N1 admitted into the ICU was 65.4% according to one study [12]. Kumar et al., in 2012, described 32 patients with ALI/ARDS with 20 cases of ARDS succumbing to illness [13]. The study period (December 2011–May 2013) began 1.5 years following the swine flu outbreak, but our hospital continued to receive cases sporadically and hence H1N1 contributed towards becoming the second most common etiology of pneumonia causing ARDS. Dengue is a major seasonal health problem in tropical countries, but the incidence of ARDS is very low, albeit with high mortality [14]. Pulmonary manifestations of dengue infection such as pleural effusion and pneumonitis are rare, but, of late, many case reports of dengue-related ARDS have been described [15]. Three cases of ARDS were due to dengue fever (*n* = 3/61; 4.91%) and all of them survived. Wang et al. evaluated 606 dengue patients and reported an incidence of 1.8% ARDS in their study [14].

Individually, *O. tsutsugamushi* was the commonest cause of ARDS. Scrub typhus related ARDS has been described mostly as case reports [16]. A 2007 study of ARDS in scrub typhus had eight cases with 25% mortality [16]. In a previous study from our institute, Vivekanandan et al. evaluated 50 cases of scrub typhus over a two-year period and showed an 8% incidence of ARDS [17]. In neighboring Tamil Nadu, 43.5% cases (*n* = 154) of scrub typhus had ARDS [18]. Acute lung injury is a rare complication of malaria with the prevalence ranging from 2.1 to 29.1% among various studies from India. ARDS can occur with infection of *Plasmodium falciparum*, *P. vivax*, or *P. ovale.* Aspiration pneumonia, concomitant Gram-negative sepsis, and fluid overload may also contribute to ARDS in malaria [19, 20]. Malaria (*falciparum*) accounted for one case of mild and moderate ARDS each, with both surviving.

Two cases of leptospirosis causing ARDS were seen, one of whom did not survive. Ninety percent of patients with leptospirosis manifest with an acute febrile illness with an excellent prognosis. Weil's disease and pulmonary manifestations are seen in the remaining 10%, many of whom progress to ARDS [21]. An ICU study from Western India revealed that leptospirosis and malaria contributed to 20% and 27.6% of ARDS cases, respectively [4]. This was the only study where tropical infections formed the majority group instead of pneumonia. We had 16 cases each of pneumonia (CAP+ VAP+ Aspiration) and tropical infections causing ARDS.

In an Indian study of 187 patients with ARDS, tuberculosis accounted for 4.9% of the total cases [22]. ARDS is generally associated with miliary TB or tuberculous bronchopneumonia [19]. Bauer et al. in 2006 had reviewed the uncommon occurrence of ARDS related to mycobacteria, viruses, parasites, and atypical pneumonias (leptospirosis, chlamydia). Scrub typhus was not discussed. Avian influenza and corona viruses were reported to cause high incidence of ARDS [10]. Jindal et al. reviewed ARDS in the tropics, where malaria, leptospirosis, strongyloidosis, enteric fever, tuberculosis, paraquat and OPC poisoning, and snake and scorpion stings were described [19]. There were no cases of animal bite or enteric fever related ARDS in our study. H1N1 infection was a seasonal occurrence. Thus the etiology of ARDS may vary depending on the geographical location, seasonal epidemics and diagnostic capabilities of the ICU/hospital; hence extrapolation of data of western countries may not be applicable in tropical developing nations. A greater proportion of tropical infections (10/16) had mild ARDS when compared to nontropical infections (8/30) which may suggest that acute lung injury is milder with tropical infections and easier amenable to therapy.

Organophosphate poisoning (OPP) can cause acute respiratory failure by mechanisms such as bronchospasm, increased bronchial secretions, aspiration pneumonia, respiratory muscle weakness, and central respiratory depression. Pulmonary edema and ARDS is also an important respiratory complication of OPP. It is possible that both patients with OPP may have had coexisting aspiration that contributed to ARDS. It has been proposed that organophosphates in the blood stream can directly cause alveolar damage and increase alveolar capillary permeability [23]. Paraquat accumulates mainly in the lung and is excreted unchanged in urine. Pulmonary complications are alveolitis acutely and fibrosis at later stages [24]. There is no antidote; albeit single lung transplantation has been found to be successful [25].

Near-hanging patients can develop various pulmonary complications such as ARDS or cardiogenic pulmonary edema due to myocardial stunning. Pulmonary complications are due to neurogenic causes or secondarily due to negative intrathoracic pressure following acute airway obstruction. Hypoxia induced hyperadrenergic states can result in translocation of blood from systemic to pulmonary circulation and can cause an increase in both pulmonary vascular resistance and increased pulmonary vascular permeability [26]. Acute pancreatitis is a common cause of ARDS in ICU patients. Increased gut permeability causes translocation of bacteria and endotoxin and activation of inflammatory mediators all of which contribute to the development of ARDS [27]. Except one case of SLE, no other inflammatory causes were identified in our study—she died of sepsis, but whether underlying vasculitis/pneumonitis existed is not known.

Mortality rates for ARDS vary widely but have, of late, shown reducing trends [28]. Most such data are from tertiary care teaching hospitals in major cities [28]. According to a Lancet review in 2007 [29], it was around 25–30%, while the mortality rates described by Phua et al. in 2008 were 30–60% [30]. The overall mortality rate of ARDS was 36% (*n* = 22) in our study, with only four patients of mild ARDS succumbing to illness. Our study had a lower mortality, possibly due to the fact that the baseline characteristics of the subjects (e.g., younger age) were different compared to the other studies and the varied etiologies of ARDS (e.g., tropical diseases) could have altered the outcome. The mortality rate in this study is in keeping with those reported by Erickson et al. Nontropical diseases had a higher relative risk (1.83) of mortality. Higher APACHE II scores (21.18 versus 7.69) were significantly related to poor outcome (*P* value of <0.001) as in other studies [31]. Patients with an indirect lung injury (score 15) had higher APACHE II scores than those with direct lung injury (score 11.6) as did moderate + severe ARDS (score 16.95) when compared to mild ARDS (score 5.30), the latter being of statistical significance (*P* < 0.001).

Limitations of our study were the small sample size, the observational nature of our study, and ventilator settings such as tidal volumes, PEEP, and plateau pressures which were not recorded. Lung Injury Score was also not recorded. Since patients were seen only once at the time of diagnosis/recruitment and again at the time of discharge/death, ventilator settings and time to organ injury could not be monitored. Hence, only SOFA scores at admission were recorded. Our focus was on the etiology of ARDS in a tropical setting where rural referrals outnumbered urban patients. Studies focusing on ARDS among rural patients were not available for comparison. Also, serological tests for atypical pneumonia like *Legionella*, *Mycoplasma*, and relevant microbiological investigations for other viruses could not be done due to dearth of such facilities in our institution which could have further given clues of patients with unknown etiology.

## 5. Conclusion 

Tropical infections commonly cause ARDS (mild-moderate), especially in the rural tropics. Other tropical medical emergencies like poisoning and near-hanging also contribute to ARDS. Tropical infections have lesser fatalities when compared to other infections. Mortality rates are low when compared to previously described studies from India. Apart from one other study, the finding of tropical infections being an important contributory cause to ARDS in ICUs of tropical and rural areas have not been previously stressed upon. This may help in creating awareness among intensivists, and institution of timely and effective antimicrobial therapy (occasionally empirical) in patients requiring critical care in the tropics, where infections such as malaria, leptospirosis, scrub typhus, and dengue fever, are endemic.

## Figures and Tables

**Table 1:** Etiology and survival of patients in our study.

Direct injury	Number of cases	Survived	Indirect injury	Number of cases	Survived
Pneumonia			Sepsis		
(1) CAP	6	6	(1) Malaria	2	2
(2) HAP	8	3	(2) MRSA	3	1
(3) H1N1	4	3	(3) *Klebsiella pneumoniae *	1	0
(4) Leptospirosis	2	1	(4) *E. coli *	1	0
(5) Scrub typhus	7	6	(5) Unidentified	5	1
(6) Aspiration	2	1	Pancreatitis	4	3
Dengue	3	3	OP poisoning	2	1
Tuberculosis	2	2	Herbicide poisoning	1	1
Toxic inhalation	1	1	Near-hanging	1	1
Near-drowning	3	3			
Paraquat poisoning	3	0			

Total	41	29	Total	20	12

MRSA: methicillin resistant *Staphylococcus aureu*; *E.coli*: *Escherichia coli*; OP: organophosphate poisoning; CAP: community acquired pneumonia; VAP: ventilator associated pneumonia.

**Table 2:** Characteristics of patients based on Berlin definition—demographics, laboratory investigations (means), complications, and hospital course.

ARDS according to Berlin definition	Mild (*n* = 24) (PaO_2_/FiO_2_ = 201–300 mmHg)	Moderate (*n* = 33) (PaO_2_/FiO_2_ = 101–200 mmHg)	Severe (*n* = 4) (PaO_2_/FiO_2_ ≤ 100 mmHg)
Age (years)	40.83 ± 13.72	41.21 ± 16.03	54.00 ± 17.79
Female sex *n* (%)	9 (37.5)	18 (54.5)	2 (50)
Duration of illness (days)	6	4	3
Heart rate (beats/min)	98.79 ± 13.28	115.73 ± 14.08	129.25 ± 9.91
Mean arterial pressure (mmHg)	90.00 ± 10.44	83.69 ± 15.72	75.83 ± 3.19
pH	7.38 ± 0.03	7.38 ± 0.06	7.19 ± 0.13
pO2 (mmHg)	87.92 ± 13.93	80.82 ± 24.29	62.50 ± 9.98
HCO3 (mmol/L)	20.25 ± 3.39	19.39 ± 4.76	11.25 ± 1.89
FiO2 (%)	42.50 ± 16.50	58.48 ± 17.77	75.00 ± 19.14
ARDS at presentation *n* (%)	22 (91.6)	21 (63.6)	3 (75)
Total leukocyte count (×10^3^/L)	10.891 ± 4.604	13.048 ± 6.238	13.475 ± 3.450
Platelets (×10^3^/L)	176.666 ± 99.589	183.121 ± 109.270	118.000 ± 91.082
ESR (mm)	59.46 ± 27.28	59.94 ± 32.22	65.00 ± 37.00
Albumin (g/dL)	3.45 ± 0.65	3.39 ± 0.89	2.75 ± 0.50
Urea (mmol/L)	13.05 ± 4.98	16.50 ± 6.64	36.88 ± 11.20
Creatinine (µmol/L)	81.04 ± 37.65	126.44 ± 75.35	316.04 ± 132.38
Glomerular filtration rate	108.13 ± 50.62	69.91 ± 33.37	22.00 ± 9.40
Glasgow coma scale (median)	14	10	8
Infectious etiology *n* (%)	18 (75)	26 (78.7)	2 (50)
Tropical infections *n* (%)	10 (41.6)	5 (15.1)	1 (25)
Direct lung injury *n* (%)	20 (83.3)	22 (66.6)	3 (75)
APACHE II score	6.54 ± 6.53	15.06 ± 8.47	28.00 ± 5.83
SOFA score	4.52 ± 2.52	7.67 ± 2.98	13.50 ± 1.29
Mechanical ventilation *n* (%)	5 (20.8)	30 (90.9)	4 (100)
Days ventilated	1.38 ± 3.18	6.48 ± 4.15	6.75 ± 3.40
Inotrope use *n* (%)	5 (20.8)	20 (60.6)	4 (100)
Duration of stay (days)	6.21 ± 2.60	9.67 ± 3.25	7.75 ± 3.30
Nonsurvival *n* (%)	4 (16.6)	14 (42.4)	4 (100)

APACHE: acute physiology and chronic health evaluation; SOFA: sequential organ failure assessment score; ARDS: acute respiratory distress syndrome; ESR: erythrocyte sedimentation rate.

**Table 3:** Univariate analysis of risk factors for mortality.

Variable	Number	Relative risk	CI	Significance
Demographics				
Age > 50 years	8/18	1.99	1.05–3.75	0.04*
Male sex	12/32	1.08	0.55–2.13	0.50
Illness duration < 1 week	20/54	1.296	0.38–4.39	0.66
Risk factors				
Smoking	8/16	1.60	0.83–3.09	0.17
Alcohol	7/14	1.56	0.82–3.06	0.21
Smoking + alcohol	7/12	1.90	1.00–3.60	0.07*
Comorbidities	14/26	2.35	1.16–4.76	0.013*
Infection	17/46	1.10	0.49–2.49	0.52
Prior hospitalization	5/7	2.26	1.23–4.18	0.038*
Clinical signs				
Temperature > 100 F	10/21	1.58	0.82–3.04	0.14
Heart rate > 100	17/42	1.53	0.66–3.55	0.20
Investigations				
pH < 7.40	14/37	1.13	0.56–2.28	0.46
TLC > 11 (×10^3^/L)	17/33	2.88	1.22–6.88	0.006*
Thrombocytopenia < 150 (×10^3^/L)	9/25	0.99	0.50–1.96	0.99
Serum albumin < 3 g/dL	13/35	1.07	0.54–2.12	0.83
Nontropical infection	32/46	3.28	0.86–12.47	0.04*
Organ dysfunction				
AKI	14/23	2.89	1.43–5.80	0.002*
Altered sensorium	20/29	11.03	2.82–43.16	<0.001*
Direct lung injury	15/45	0.76	0.38–1.52	0.32
ARDS	38/61	1.92	1.34–2.75	0.001*
SGOT > 3 times elevation	19/50	1.39	0.49–3.89	0.38
SGPT > 3 times elevation	20/54	1.29	0.38–4.39	0.50
APACHE II score > 20	14/61	5.36	2.81–10.22	<0.001*
Hospital course				
ARDS after admission	10/15	2.55	1.39–4.67	0.004*
Ventilation > 7 days	11/17	2.58	1.39–4.81	0.005*
Inotrope use	20/29	11.03	2.81–43.16	<0.001*
CPR	18/19	17.19	2.54–116.11	<0.001*

TLC: total leukocyte count; AKI: acute kidney injury; ARDS: acute respiratory distress syndrome; SOFA: sequential organ failure assessment score; APACHE: acute physiology and chronic health evaluation; SGOT: serum glutamic oxaloacetic transaminase; SGPT: serum glutamic-pyruvic transaminase; CPR: cardiopulmonary resuscitation. *indicates significant *P* value <0.05
